# Prevalence of Calf Mortality in Ethiopia: A Systematic Review and Meta-Analysis

**DOI:** 10.1155/2021/6677986

**Published:** 2021-11-16

**Authors:** Ephrem Tora, Mesfin Shrube, Tamirat Kaba, Wasihun Seyoum

**Affiliations:** Arba Minch University, College of Agriculture Sciences, Department of Animal Sciences, P.O. Box 21, Arba Minch, Ethiopia

## Abstract

**Background:**

Calf mortality has been a major cause of economic losses in the dairy industry in Ethiopia. The condition results in a significant drop in the number of replacing heifers and bulls for sustainable dairy business. Reports on calf mortality with a wide range of prevalence are rising in the country; however, a pooled prevalence of this condition has not been established. Thus, this systematic review and meta-analysis aimed to quantitatively estimate the pooled prevalence of calf mortality in Ethiopia.

**Methods:**

Meta-analysis was carried out to obtain the pooled prevalence of calf mortality in Ethiopia. A comprehensive literature search was carried out on PubMed, African Journals Online, CAB, Web of Science Direct, and Google Scholar. Eligible studies were selected based on predefined inclusion and exclusion criteria. Moderators such as the study area, breed of calf, study design, agroecology, and year of study article published were used as a milestone of data extraction. The random-effect model was used to estimate pooled prevalence. Publication bias and the variation in prevalence estimates attributed to heterogeneity were also assessed.

**Results:**

Twenty-five original research papers on the prevalence of calf mortality in various parts of Ethiopia were included. The reported prevalence of calf mortality was between 0.9% and 37%. The pooled prevalence of calf mortality in the country was 14.79%, and the pooled calf mortality estimate across studies for the entire period regarding 1991 to 2000, 2001 to 2010, 2011 to 2016, and 2017 to 2020 was 26.54%, 17.03%, 14.21%, and 11.23%, respectively. Analysis of study subgroups and location revealed significant variations in prevalence. High heterogeneity was observed in the pooled estimates and even after the subgroup meta-analysis. The funnel plots and Egger's regression asymmetry coefficient (*b* = −1.0434) (95% CI = −1.49, −0.59; *p* value of 0.012) did suggest the presence of publication bias. There was also an indication of missing studies that could be incorporated by Duval and Tweedie's trim and fill method where they might fall on a funnel plot and visualize them in an attempt to increase the plot's symmetry. Analyses also suggest that calf breed, sample size, and study location are likely to be moderators of calf mortality prevalence in Ethiopia.

**Conclusion:**

This finding shows that calf mortality is widespread and could result in considerable economic losses for the dairy industry in Ethiopia. Inevitably, a significant reduction in calf mortality prevalence has been observed in recent years since 2010, but the reduction has not yet reached an economically tolerable level. Calf breed susceptibility contributed to the high prevalence. Therefore, interventions for increasing calf health and performance should be focused on minimizing calf mortality on farm and animal levels.

## 1. Introduction

Ethiopia has the largest livestock population, being the first in Africa and the tenth in the world [1, 2]. The livestock sector of this huge population plays an important role in the national economy as well as in the socioeconomic development of millions of rural smallholder farmers as income, employment generation, and poverty alleviation [3]. Moreover, the sector contributes to 15% of export earnings and currently supports the livelihoods of 80% of the rural population [1, 4].

The dairy industry being part of the livestock production unit has been recognized as one of the most prominent industries in the pursuit to attain food security and good welfare [5]. In Ethiopia, there is a growing trend in the development of market-oriented urban and periurban dairy farming, which is becoming an important supplier of milk and milk products. Small scale intensive production systems are the primary source of income for urban and periurban poor communities by maintaining supply of milk and milk products to the communities and continue to be in the future [6, 7]. This sector generates over 80% of milk, which makes it a key component of the dairy segment and its future development [5].

The expansion of the dairy segment in Ethiopia has a considerable prospective opportunity for dairy producers' food and nutrition security [8]. However, dairy production is severely affected by several constraints. Among the constraints, calf diseases and preweaning mortality are the major ones that determine successful rearing of replacement stock [9, 10]. Calf mortality incurs a great economic loss to dairy producers. This arises from decreased lifetime productivity, death loss, and survivorship. It also causes the loss of genetic material for herd improvement and decreases the number of dairy heifers available for herd replacement and expansion [11], leading to a shortage of dairy replacement heifers [9, 12, 13]. Since replacement heifers are of supreme importance to the total profitability of dairy farms, dairy heifers are basic stocks for future herds [14].

Over the years, a number of researchers have reported the occurrence of calf mortality in Ethiopia. According to the studies, the apparent prevalence of calf mortality estimate falls within the range of 0.9% and 37.3% based on observational studies [6, 9, 10, 15–17]. This shows that the current apparent prevalence range includes 20% calf mortality rate, which is estimated to reduce net farm profit by 38% [18]. Also, calf mortality is ranked next to mastitis as the second main problem for dairy production in Ethiopia [19]. Besides, the aforementioned studies have shown that different factors such as the breed of calf, agroecology, and geographic locations contribute to the variations of apparent prevalence of calf mortality. However, to date, there has been no quantitative synthesis of the reports conducted to describe the spatial-temporal distribution and the most important calf and environmental risk factors influencing the occurrence of calf mortality in the country. Such information could be of significant importance in the development of feasible intervention measures aimed at reducing the burden of deaths and productivity losses related to deaths. Thus, this systematic review and meta-analysis was aimed at (i) estimating a pooled prevalence of calf mortality and (ii) identifying the relevant predictors that could possibly dictate the observed pooled prevalence in Ethiopia based on data from a number of studies.

## 2. Material and Methods

This meta-analysis study was conducted based on the Preferred Reporting Items for Systematic Reviews and Meta-Analyses (PRISMA) statement [20]. The PRISMA checklist was used to ensure the inclusion of relevant information in the analysis. The outcome of interest was the prevalence of calf mortality across the country, Ethiopia.

### 2.1. Study Area Description

The systematic reviews and meta-analysis was conducted in Ethiopia, a country situated in the horn of Africa located between 3°00′–15°00′ N latitude and 32°30′–48°00′ E longitude. Ethiopia has a land area of 1.04 million km^2^ and a population of 116 million [21], the second most populous nation in Africa next to Nigeria [22]. Ethiopian climate is suitable for agriculture, and it is also a home for an estimated 60.4 million heads of cattle [2]. Ethiopia has a diverse topography, which forms the basis for different agroclimatic zones. The area located above 2300 m above sea level (m.a.s.l.) is considered highland, which is surrounded by a temperate transition zone between 1500 and 2300 m.a.s.l., called midland, while the area with an altitude below 1500 m.a.s.l. is classified as lowland [23].

### 2.2. Literature Search Strategy

**Figure 1:**
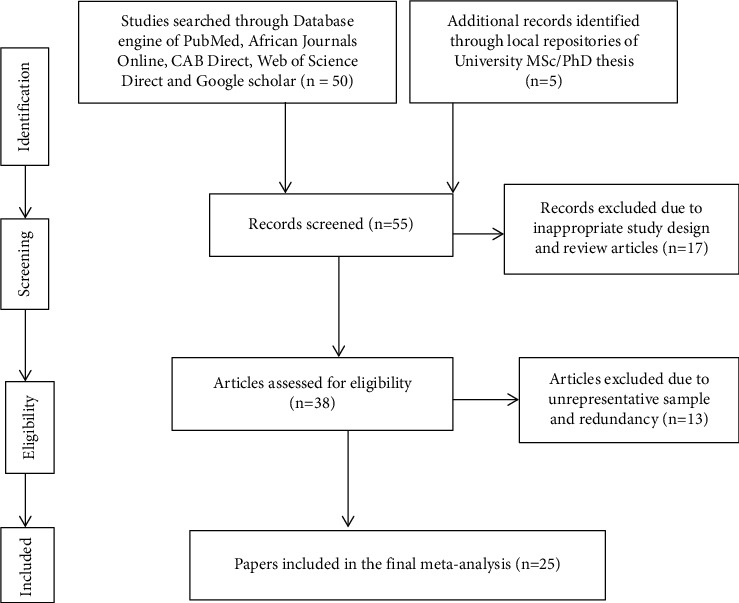
Schematic representation of the literature selection procedure for the systematic review of calf mortality prevalence in Ethiopia that depicts excluded and included studies.

A comprehensive literature search on calf mortality in Ethiopia was executed from February 2020 to April 2020. Electronic databases, such as PubMed, CAB direct, African Journals Online, Google Scholars, and Web of Science Direct, were used. Besides, local institutional repositories were searched to retrieve unpublished MSc theses. The following Medical Subjects Headings (MeSH) terms were used in the electronic database search engine: calf, mortality, calf death, and Ethiopia. These terms were reorganized to phrases as adjacent as “calf mortality in Ethiopia,” “prevalence of calf mortality in Ethiopia,” and “prevalence and Ethiopia.” Studies that reported on calf mortality, including in different calf breeds which met eligibility criteria, were fitted to the final meta-analysis. Study screening strategy and exclusion reasons are presented in Figure 1.

### 2.3. Inclusion and Exclusion Criteria

All Articles that have studied the prevalence of calf mortality in Ethiopia so far were downloaded and added to the Mendeley reference manager. The inclusion and exclusion criteria were set for the eligibility of studies before starting review processes. Inclusion criteria were defined regarding the relevance of the articles to the meta-analysis hypothesis of our interest if (i) it was published in English, (ii) it was an observational study that reports the prevalence of calf deaths, and (iii) it was published article or MSc thesis in university repositories that reported the prevalence of the mortality only in calves of different breeds from 1991 to 2020. Otherwise, studies were excluded if the titles and abstracts were not relevant to the outcomes of interest or did not fulfill the eligibility criteria. Duplicates were also strictly checked and excluded. In this study, calves are defined as the age group of young cattle from birth to nine months of age. Articles that met the above criteria were deliberated for the final meta-analysis and systematic review. Titles were checked twice in both excluded and included databases of the Mendeley reference manager before the start of the data extraction process to avoid missing a valuable report independently. Those relevant papers were maintained, and their results were worked out to a preprepared data extraction Microsoft excel sheet.

### 2.4. Data Extraction

The data extraction template was developed on plausible predictors, which were consistently reported in most of the selected published articles. The predictor dataset includes study location, sample size, number of deaths, calf breed, agroecology, study design, author's name, and year of publication/study. Authors were engaged fully in data extraction independently based on plausible predictors. The template was further reviewed and enriched. Finally, the selected articles and the dataset generated were cross-checked, and ambiguities were ruled out by group discussions.

### 2.5. Literature Bias Assessment

Articles were evaluated to observe the within-study bias across literature for quality assessment because objectivity and consistency were a priority. Publication bias was assessed by both visual examination of funnel plot and Egger's regression asymmetry test [24]. Meta-regression was used to investigate factors potentially contributing to the between-study heterogeneity. Duval and Tweedie's nonparametric “fill and trim” linear random method was used to calculate the unbiased estimates [25].

### 2.6. Data Analysis

Descriptive summary statistics were calculated to determine the total number of sampled calves under study and the range of prevalence estimates in different calf breeds. The data were transformed into a logarithmic scale to normalize distribution using the formula ES=  log_*e*_(*p*/(1 − *p*)), where ES is the effect size, log_*e*_ is the natural logarithm, and *p*is the study level estimate. The pooled estimate was computed from transformed values by back transforming the transformed values into original units of proportions, as per formula *p*=*e*^logit^/(*e*^logit^+1). Forest plot in the random-effect model was used to present the effect size and associated weight for each report included in the review.

Heterogeneity between studies was evaluated through Cochran's *Q* test (reported as *p* value), inverse variance index (*I*^2^), known as the Higgins statistic, was used to compute the observed proportion of variability attributed to the heterogeneity of studies, and meta-regression was conducted to assess the level of variation among the predictors [26]. Meta-regression analysis was done separately for each moderator included in the study. The moderators included were categorical variables such as sample size category, year of study, study location, calf breed, study design, and agroecology. Those variables had significant values and were retained in the final multivariable analysis. Forest plot was used to depict the level of existing difference beyond chance in Cochran's statistics (*Q*). The standard variation between the studies was also computed (*τ*^2^). Multicollinearity assessment for predictors was made using Kruskal Gamma statistics in univariable regression, and predictors with Gamma value between ±0.6 were subjected to multiple meta-regressions [27]. This approach allowed us to identify the explainable proportion of the heterogeneity (*I*^2^) attributed to predictors that have a significant statistical association (*p* < 0.05). Subgroup analysis was conducted to get pooled estimate for the predictor's category. Results with *p* ≤ 0.05 were considered statistically significant. The R Core Team program [28] was used to analyze the data.

## 3. Results

### 3.1. Characteristics and Quality of Studies

Fifty-five published papers and MSc theses were retrieved for calf mortality during the literature search period. Among the retrieved articles, only 25 reports met the inclusion criteria for the systematic review and meta-analysis (Figure 1). The dataset was composed of 30 observations while splitting up for calf breeds (Table 1). However, about 30 reports were dissimilar, having unrepresentative sample size, inappropriate study designs, and different agroecology with the disproportionate sample; thus, they were excluded together with the lack of data on other required predictors. This between-studies variability nature reduced substantially our datasets to be included in the meta-analysis. The studies include papers that were conducted in the period from 1991 to 2020. The total number of calves enrolled and died in the studies was 13,762 and 2,571, respectively. The sample size ranged from 30 to 1829 calves (mean: 529; standard error (SE): ±90). The range prevalence of calf mortality documented in the literature varied from 0.9% to 37.3% (mean: 17.4%; standard error (SE): ±1.8%) [6, 9, 10, 15–17].

### 3.2. Geographical Distribution of Included Studies in Ethiopia

**Figure 2:**
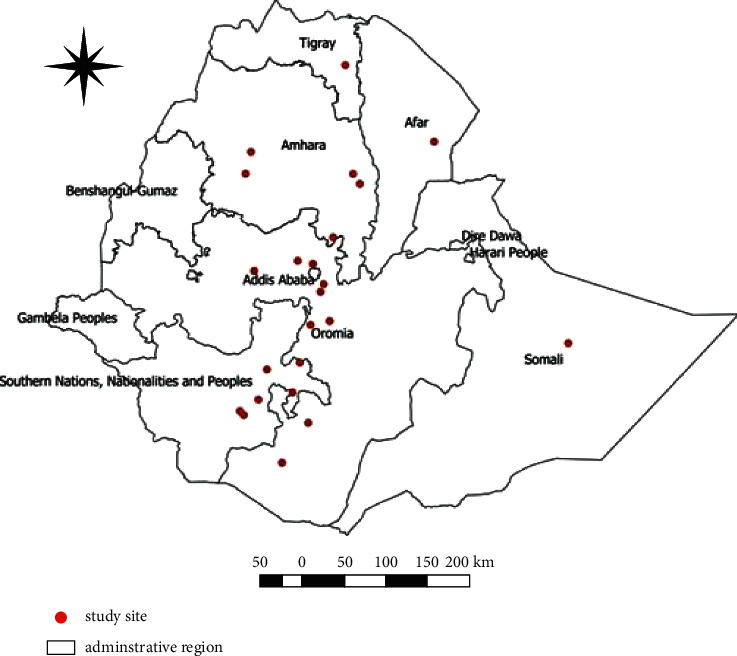
Observed spatial distribution of calf mortality in Ethiopia.

The reports were collected from studies conducted in six regional states, namely, Amhara, Afar, Oromia, South Nations Nationalities and Peoples Region, Somali, and Tigray, between 1991 and 2020. However, these regions were categorized into four parts based on the distance and direction from the capital city of the Ethiopia of the country, namely, central, northern, northwest, and southern Ethiopia, to manage data easily for available valid reports on calf mortality. Central Ethiopia is a location having a radius of 150 kilometers from Addis Ababa, the capital of Ethiopia. Most of the studies were concentrated in central Ethiopia. The apparent prevalence varied from study location to location ranging from 0.9% to 37.3% (Table 1). A total of 48 survey districts were identified from the 25 studies. However, five sites were not included in the map due to the lack of a unique location identifier. Some of the studies were not mapped on the spatial distribution due to the same location identifier on the same area. Spatial distribution and observed prevalence by location are depicted in Ethiopia (Figure 2). Some parts of the country, namely, central, southern, and northwest Ethiopia, contain a large number of survey locations, while northern Ethiopia has only a few calf mortality studies.

### 3.3. Meta-Analysis

**Figure 3:**
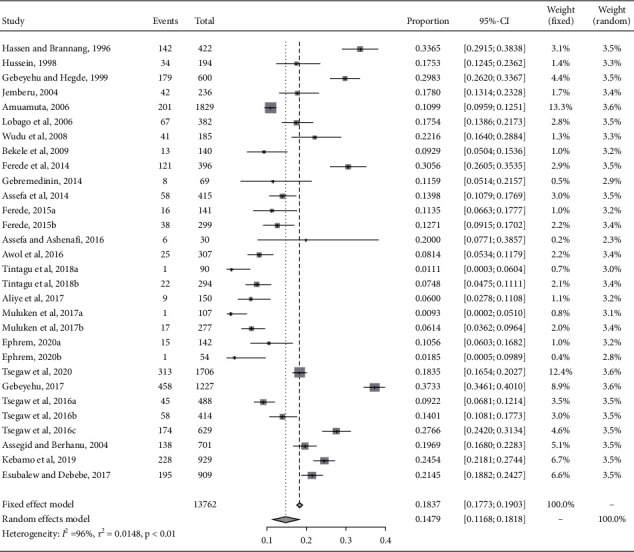
Forest plot visualizing the varying calf mortality prevalence reported for each study included publication in the meta-analysis. Weightage given to each included publication by both RE and FE models has been shown for rigorous comparison.

The pooled prevalence estimate calculated from a random-effect (RE) meta-analysis and forest plot is depicted (Table 2; Figure 3). Accordingly, the pooled estimate was 14.79% with 95% CI (11.68, 18.18). As of the variation between studies, RE meta-analyses were carried out using the total sample size and the number of deaths (effect size and standard error of effect size). The meta-analysis indicated that between-study variability was high (*τ*^2^=0.416, heterogeneity *I*^2^= 96.76% with heterogeneity chi-square = 777.6, and *p* value of 0.0001). The meta-analysis and comparison to the RE model are graphically summarized in a forest plot (Figure 3).

### 3.4. Meta-regression

#### 3.4.1. Univariable Meta-regression

The individual variable contribution to the explainable proportion of reported heterogeneity was observed in the analysis in order to determine the effect of study level covariates on the estimates of cumulative prevalence. As shown in Table 3, the proportion of each predictor variable's effect on heterogeneity (*I*^2^) ranged from 0% to 11% in the RE model. Under the RE model, the highest value of *I*^2^ was observed for the year of study and sample size, while study design, study location, and agroecology exhibited no effect on heterogeneity (*I*^2^ = 0%). Ultimately, the *I*^2^ values range between 0 and 1, but when the sampling error yielded a proportion value outside the specified range, the calculated values were set to either 0 if negative or 1 when it was above 1 [41].

#### 3.4.2. Multivariable Meta-regressionx

In the multivariable meta-regression, year of study, calf breed, and sample size were considered with their respective *p* value below 0.25 in univariable meta-regression. All the three variables had a significant effect on prevalence but the model, however, accounted for only 30.2% of the explainable proportion of the between-study variance (*τ*^2^ unexplained = 0.3044). According to the multivariable model, the prevalence in crossbred calves was over 64.7% higher than that of the prevalence in local breeds (Table 4). In the subgroup analysis presented, the highest prevalence (17.5%) was noted in central Ethiopia, while the lowest (12.1%) was in northwest Ethiopia (Table 2). The multivariable meta-regression model showed pooled prevalence estimate of calf mortality increase by 77.6% from 1991 to 2000 when compared to 2017 to 2020 (Table 4).

### 3.5. Sensitivity Analysis

**Figure 4:**
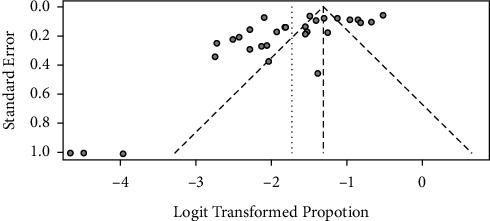
Funnel plots of the standard error by transformed prevalence estimates.

The funnel plots (Figure 4) and Egger's regression asymmetry coefficient (*b* = −1.0434) (95% CI = −1.49, −0.59; *p* value of 0.012) did suggest the presence of publication bias, and there was also an indication of theoretical missing study that was incorporated by Duval and Tweedie's trim and fill method. This evidence of publication bias suggests that the RE model was more appropriate for these data.

## 4. Discussion

This is the first meta-analysis of pooled prevalence of calf mortality in Ethiopia, reporting pieces of literature from 1991 to 2020 that were obtained through analysis of a systematic review and meta-analysis that pooled 25 published articles and Masters theses on calf mortality of dairy cattle. Therefore, it is vital to communicate the status of pooled calf mortality to estimate the total effect, to set out control strategies for successful rearing of the calves and replacement heifers. However, the number of studies to be included in the final meta-analysis had been substantially diminished due to heterogeneous literature, inappropriate study designs, and unrepresentative sample size, as well as lack of data on the required variables and related other factors.

The executed meta-analysis of pooled prevalence of calf mortality varied over time, with the lowest levels since 2016. This could be associated with easy access to veterinary service and current increment of public awareness [6, 8, 12, 29]. In Table 2, the pooled prevalence of calf mortality in the RE model of the current report was 14.79% (95% CI; 11.68–18.18%). This finding is significantly higher than the economically tolerable limit of mortality magnitude (3–5%) that could be achieved through good calf management at an economically tolerable level [18, 42, 43]. This suggests that calf mortality is the major constraint that could result in a serious economic crisis in the Ethiopian dairy sector. Moreover, this finding is alarming that Ethiopia, despite having Africa's largest livestock number and accounting for one-tenth world's livestock population, has an urgent and yet unmet need for dairy production and replacement herd requirements. Therefore, there should have a mandatory approach to reduce calf mortality so as to improve profitability in Ethiopia.

In general, this study showed a decreasing trend of prevalence of calf mortality as one moves from central parts of the country to the margins in all geographic directions and from accessible regional areas to more distant places where subsistence extensive livestock husbandry practices dominate (Table 2). The calf mortality prevalence appeared highest in central parts of Ethiopia that could be due to comparatively long history of intensive modern dairying with records which was started when Ethiopia received the first batch of exotic breeds in the early 1950s from the United Nations Relief and Rehabilitation Administration (UNRRA) in Addis Ababa and its surroundings [44, 45]. Also, this may be due to the fact that most dairy producers, of government and private commercial, are found in this area which surrounds the capital city of Ethiopia and mostly attract the attention of researchers and funders revealing the real calf health problems. Moreover, the prevalence was higher in studies documented in the central part of the country due to the rearing practices of exotic breeds, which are susceptible to the tropical environment and poor management practices.

Even though the first attempt to dairy development by the support of the UNRRA program gave an opportunity to the start of dairy production and maintenance of animals for the breeding program, the improvement on the milk production and replacement dairy herd availability was low due to mortality conditions (and still stagnant), causing a shortage of replacement herd [6, 40, 46]. Our results also disclosed the high pooled prevalence of calf mortality in dairy calves across parts of the country. The high calf mortality may be related to husbandry problems and no intervention like vaccination of calves and other biosecurity measures [1, 7, 37]. This increased calf mortality has contributed to large economic loss and will continue to grow if no intervention measures are implemented. It is therefore beyond the economically tolerable level and needs further identification of potential risk factors for calf mortality, thus setting out strategies to control causes of calf mortality.

Many factors are supposed to be linked to the prevalence of calf mortality. Regarding calf breeds (crossbred versus local breeds), the present meta-analysis (RE model) shows prevalence to be higher in crossbred (17.8% (95% CI: 13.2, 21.6)) than in indigenous breeds (10.5% (95% CI: 6.87, 15.7)) (Table 2). This finding is supported by several published studies on the influence of breed on genetic susceptibility to various diseases, which showed that the native breed of calf is more resistant to death than the exotic breed [6, 9, 47]. However, rigorous investigations of the true differences in susceptibility among varied calf breeds to calf mortality would be necessary due to the heterogeneity of observed studies for a rational evidence-based formulation of strategies to prevent or reduce calf death in Ethiopia.

Despite the lack of significant difference between agroecological categories for the prevalence of calf mortality, the highest pooled estimate was observed in lowland, which was 20.0%, followed by 12.5% in highland and 15.6% in midland (Table 2). When the overall status of calf mortality is considered from an agroecological perspective, the findings clearly highlighted the need for attention regardless of the agroecology in which dairy calves are raised. The situation in the lowland of the country, however, should be viewed differently as the numbers of reports were only two, and it is difficult to consider them representative for the lowland agroecology. In fact, agroecology has a biological explanation as a predisposing factor to causative agents that precipitate calf mortality; therefore, the findings could reveal an interesting spatial pattern that should be further investigated. Moreover, calves graze together with their dams usually after two-month age in lowland agroecology, which is almost extensive production, and this may precipitate calves more to diseases and, in turn, to mortality.

In this multivariable meta-regression, the model was responsible for 30.2% of the heterogeneity between studies, signifying that additional factors which were out of the model were contributors to calf mortality prevalence. These factors may be such as calf age, sex, production system, and herd size that were not denoted with enough frequency in these articles included in the systematic review to be subject to a rigorous meta-analysis [9, 34, 39]. Henceforth, prospect studies should attempt to comprehend how these factors contribute to overall calf mortality prevalence. We need to insist also that the findings of a meta-analysis must be applied with caution due to limitations inherent in publication bias and heterogeneity. Besides concern of consistency, certain studies suffer from the shortage of clear description of the study area with regard to animal husbandry practices, georeferencing, calf breed description whether cross or local, and presence or absence of other coexisting animal species, of informal culling practice, and of farm hygiene. Further, our review is limited to published articles written in English and MSc theses loaded in repositories, subjecting our analysis to publication bias (Figure 4). This potential bias is supported by the significant result of Egger's regression test [48].

Calf mortality can be caused by infectious and noninfectious agents, which might be controlled strategies supported by policy regulations in several countries. These general control strategies include general management before birth; care of calf after birth; feeding of calves after birth; and deworming [49]. Applying these control strategies could be effective and result in reducing calf mortality at an economic level and lowering individual and herd-level prevalence [42]. This policy-led strategy can be practically possible in developing countries like Ethiopia if the emphasis is given to these practices by owners and the use of rational veterinary services. Alternatively, practices like rearing of heifers in designated centers and distributing them will reduce calf loss due to poor husbandry practices. However, future application should be on the optimization of comprehensive control strategies that could be implemented in Ethiopia.

## 5. Conclusion

The pooled result of 14.79% calf mortality in the present meta-analysis conducted on 25 publications indicated higher and above the economically tolerated level of calf mortality prevalence in Ethiopia. This shows calf mortality as an important cause of losses to the farmer as well as the economy of the country. However, the prevalence of calf deaths has shown a significant reduction since 2016. The prevalence was higher in studies documented in the central part of the country due to the rearing practices of exotic breeds, which are susceptible to the tropical environment and poor management practices. To this end, the implementation of the comprehensive control measures for the prevention of calf deaths should be adopted, provided that the future of the livestock economy depends on the calves adopting simple management practices such as ensuring adequate intake of colostrum in early life, proper housing, good hygiene to minimize disease transfer, and affording clean drinking water. In addition, appropriate feeding protocols to encourage early rumen development and proper animal healthcare can lead to improved calf performance and can reduce calf mortality.

## Figures and Tables

**Table 1:** Study reports included in a meta-analysis of calf mortality in calf breeds in Ethiopia.

Author and study year	Calf breed	Study site/reported from	Agroecology	Study design	Total	APP (%)
Aliye et al., 2017 [29]	Local	Southern Ethiopia	High and midland	Cohort	150	6.00
Amuamuta et al., 2006 [15]	Local	Northwest Ethiopia	Midland	Cohort	1829	10.9
Asmare and Kiros, 2016 [30]	Cross	Southern Ethiopia	Midland	Cohort	30	20.0
Assefa et al., 2015 [31]	Cross	Southern Ethiopia	Midland	Cohort	415	13.9
Assegid and Berhanu, 2004 [32]	Cross	Central Ethiopia	Highland	C/sectional	701	19.7
Awol et al., 2016 [3]	Cross	Northern Ethiopia	Highland	C/sectional	307	8.14
Megersa et al., 2009 [17]	Cross	Southern Ethiopia	Midland	Cohort	140	9.29
Ephrem, 2020 [12]	Cross	Southern Ethiopia	High and midland	Cohort	142	10.5
Ephrem, 2020 [12]	Local	Southern Ethiopia	High and midland	Cohort	54	1.85
Sisay and Dessie, 2017 [33]	Cross	Northwest Ethiopia	Highland	Cohort	909	21.4
Ferede et al., 2014 [16]	Cross	Northwest Ethiopia	High and midland	C/sectional	396	30.5
Ferede, 2015 [34]	Local	Northern Ethiopia	High and midland	Cohort	141	11.3
Ferede, 2015 [34]	Cross	Northwest Ethiopia	High and midland	Cohort	299	12.7
Gebeyehu and Hegde, 2003 [35]	Cross	Northern Ethiopia	Highland	C/sectional	600	29.8
Gebeyehu, 2017 [34]	Cross	Central Ethiopia	Midland	Cohort	1227	37.3
Gebremedihin, 2014 [16]	Cross	Central Ethiopia	Midland	Cohort	69	11.6
Hassen and Brannang, 1996 [36]	Cross	Central Ethiopia	Midland	C/sectional	422	33.6
Hussein, 1998 [35]	Cross	Central Ethiopia	Midland	C/sectional	194	17.5
Jemberu, 2004 [37]	Cross	Central Ethiopia	Midland	C/sectional	236	17.8
Kebamo et al., 2019 [38]	Local	Southern Ethiopia	Midland	Cohort	929	24.5
Lobago et al., 2007 [39]	Cross	Central Ethiopia	Midland	C/sectional	382	6.14
Muluken et al., 2017 [1]	Cross	Northwest Ethiopia	Midland	C/sectional	277	17.5
Muluken et al., 2017 [1]	Local	Northwest Ethiopia	Midland	C/sectional	107	0.9
Tintagu et al., 2018 [6]	Cross	Central Ethiopia	Highland	C/sectional	294	7.48
Tsegaw et al., 2016a [40]	Local	Northwest Ethiopia	Midland	C/sectional	488	9.22
Tsegaw et al., 2016b [40]	Local	Central Ethiopia	Lowland	C/sectional	414	14.1
Tsegaw et al., 2016c [40]	Local	S. eastern Ethiopia	Lowland	C/sectional	629	27.6
Tsegaw et al., 2020 [10]	Cross	Central Ethiopia	High and midland	C/sectional	1706	18.3
Wudu et al., 2008 [9]	Cross	Central Ethiopia	Midland	Cohort	185	22.1
Tintagu et al., 2018 [6]	Local	central Ethiopia	Highland	C/sectional	90	1.11

APP: apparent prevalence. Total: sample size. C/sectional: cross-sectional.

**Table 2:** Pooled prevalence estimates of calves' mortality, stratified by subgroups.

Predictors	Number of studies	Pooled prevalence of calf mortality			Heterogeneity (*I*^2^)	
		Sample size	Cases	Prevalence (95% CI) (RE model)	*I* ^2^ (%)	*p* value (Cochran's *Q*)
Overall prevalence	30	13762	2571	14.8 (12.4–19.2)	96.79	<0.01
Study location						
Central Ethiopia	12	5920	1324	17.5 (17.4–24.1)	96	<0.01
Northern Ethiopia	3	1048	220	14.8 (7.30–27.7)	97	<0.01
Southern Ethiopia	8	2489	504	13.5 (8.51–20.8)	91	<0.01
Northwest Ethiopia	7	4305	618	12.1 (7.43–19.0)	96	<0.01
Study design						
Cross-sectional	14	7243	1379	15.1 (11.2–20.2)	96	<0.01
Cohort	16	6519	1287	14.5 (10.3–19.9)	97	<0.01
Agroecology						
Midland	15	6930	1361	15.6 (11.2–21.2)	97	<0.01
High and midland	7	2888	513	13.5 (7.82–22.3)	95	<0.01
Highland	6	2901	560	12.5 (7.45–20.2)	92	<0.01
Lowland	2	1043	232	20.0 (8.55–40.2)	96	<0.01
Calf breed						
Indigenous	10	4831	734	10.5 (6.87–15.8)	95	<0.01
Cross	20	8931	1932	17.1 (13.2–21.6)	95	<0.01
Year of study						
1991–2000	3	1216	355	27.1 (19.4–35.4)	95	<0.01
2001–2010	8	4798	851	18.7 (13.6–24.4)	95	<0.01
2011–2016	10	4498	741	14.2 (10.5–18.3)	91	<0.01
2017–2020	9	3250	719	8.46 (2.34–17.6)	98	<0.01
Sample size						
<200	7	934	121	11.2 (6.84–17.6)	79	<0.01
200–384	6	1339	163	11.3 (6.70–18.5)	85	<0.01
>384	17	11489	2382	17.7 (13.6–22.8)	97	<0.01

**Table 3:** Proportion of the between-study variance explained (*I*^2^) by each variable considered in meta-regression on the prevalence of calf mortality in Ethiopia.

Predictors	Proportion (*I*^2^) (%)	*p* values (RE)
Study design	0.00	0.8604
Study location	0.00	0.4234
Year of study	11.0	0.0507^*∗*^
Calf breed	0.53	0.0511^*∗*^
Sample size	8.76	0.1122^*∗*^
Agroecology	0.00	0.7532

*Note.* The proportion of the effect of predictors on heterogeneity. All variables had *p* < 0.01 in the FE model. Significant results of individual predictors subjected to multivariable meta-regression. ^*∗*^Significance.

**Table 4:** Multivariable meta-regression model on prevalence of calves mortality in Ethiopia (*I*^2^ = 30.2%; *N* = 30 reports).

Predictors	Category	Prevalence (95% CI)	*N*	Coefficient	*p* value
Calf breed	Local	10.54 (6.87–15.83)	10	Reference	
	Cross	17.01 (13.22–21.62)	20	0.6054 (0.0725, 1.1384)	0.0219

Sample size	<200	11.16 (6.84–17.6)	7	Reference	
	200–384	11.32 (6.78–18.5)	6	−0.7498 (−1.3946, -0.1050)	0.0227
	>384	17.78 (13.65–22.83)	17	0.6641 (0.0868, 1.2414)	0.0242

Year of study	2017–2020	8.46 (2.34–17.67)	9	Reference	
	2011–2016	14.18 (10.50–18.34)	10	0.4406 (−0.2005, 1.0818)	0.1780
	2001–2010	18.72 (13.65–24.34)	8	0.7651 (0.1014, 1.4288)	0.0239
	1991–2000	27.07 (19.45–35.43)	3	1.2415 (0.3677, 2.1153)	0.0054

## Data Availability

All data analyzed are included in this published article.
